# Psychometric evaluation of the Turkish version of the Fear of Crime Scale

**DOI:** 10.1186/s40359-026-05110-3

**Published:** 2026-07-02

**Authors:** Mehtap Genç, Emre Çiydem, Muhammed Ferit Duman, Canan Bozkurt Duman

**Affiliations:** 1 Department of Mental Health and Psychiatric Nursing, Faculty of Health Sciences, Karamanoğlu Mehmetbey University, Karaman, 70100 Türkiye; 2 Department of Mental Health and Psychiatric Nursing, Faculty of Nursing, İstanbul University, İstanbul, Türkiye; 3 Department of Social Work and Counseling, Vocational School of Health Services, Bandirma Onyedi Eylul University, Balıkesir, Türkiye; 4 Department of Internal Medicine Nursing, Faculty of Health Sciences, Bandirma Onyedi Eylul University, Balıkesir, Türkiye

**Keywords:** Fear of crime, Validity, Reliability

## Abstract

**Background:**

Crime is a multidimensional phenomenon that threatens social order and varies across contexts. In Türkiye, crime patterns and their changes over time play a key role in shaping fear of crime. However, measurement tools that directly and multidimensionally assess fear of crime in the Turkish context remain limited.

**Aim:**

This study aims to evaluate the psychometric properties of the Turkish version of the Fear of Crime Scale.

**Methods:**

The sample of this cross-sectional methodological study consisted of 849 adult individuals living in Turkey who met the inclusion criteria. Content validity was assessed using the Content Validity Index based on the Davis technique. Construct validity was examined through confirmatory factor analysis, and convergent and discriminant validity were evaluated using Pearson correlation analyses. Reliability was assessed using Cronbach’s alpha coefficient, item-total correlations, and test-retest methods.

**Results:**

The overall Content Validity Index value was 0.981, and item-level CVI values ranged between 0.86 and 1.00. Confirmatory factor analysis confirmed the original single-factor structure with 10 items; standardized factor loadings ranged from 0.717 to 0.919. Model fit indices were determined as χ²/df = 4.90, GFI = 0.92, AGFI = 0.87, RMSEA = 0.08, IFI = 0.96, TLI = 0.93, NFI = 0.91, and SRMR = 0.05; AVE and CR values were calculated as 0.69 and 0.95, respectively. Cronbach’s alpha coefficient was 0.94, and item-total correlations ranged from 0.616 to 0.842. In convergent and discriminant validity analyses, the scale showed a positive correlation with the Fear of Crime Scale (*r* = 0.532, *p* < 0.001) and a significant negative correlation with the Life Satisfaction Scale (*r* = − 0.240, *p* = 0.041). Test-retest reliability was calculated as ICC = 0.927 (95% CI: 0.901–0.960, *p* < 0.001).

**Conclusion:**

The findings indicate that the Turkish Fear of Crime Scale is a valid and reliable measurement tool for assessing fear of crime among adult individuals.

**Supplementary Information:**

The online version contains supplementary material available at 10.1186/s40359-026-05110-3.

## Introduction

As a consequence of living together, crime is a universal phenomenon that has existed in all societies since the beginning of humanity. At the same time, it represents a fundamental social issue that threatens social order and requires a multidisciplinary approach to be addressed. Different disciplines such as law, anthropology, sociology, and psychology examine crime from diverse perspectives; in particular, the disciplines of psychology and sociology address crime within the framework of individual and social behavior patterns, as well as the cognitive, emotional, and social processes that shape these behaviors [1, 2]. In this context, crime can be considered not only a legal violation but also a complex and multidimensional behavior that emerges from the interaction between the individual and their environment.

When the data from the Turkish Statistical Institute (TÜİK) are examined, it is observed that the number of convicted individuals entering correctional institutions increased from 168,726 in 2015 to 281,605 in 2019, indicating an approximate 67% rise [3]. Indeed, according to 2024 data from the Ministry of Justice of the Republic of Türkiye, 15.8% of convicted individuals were sentenced for theft, 12.0% for intentional injury, and 10.1% for drug-related offenses; together, these three categories account for approximately 38% of all crimes [4]. When violent crimes are examined, it is observed that intentional injury offenses are approximately 6–7 times more prevalent than intentional homicide. This indicates that crime in Türkiye is more concentrated in high-frequency but relatively low-lethality offense types, and that it exhibits a clustered pattern within certain crime categories. While the incarceration rate in Türkiye is 319 per 100,000 population [3], the intentional homicide rate—approximately 3–5 per 100,000—indicates that Türkiye has a profile close to the European average in terms of lethal violence and a lower risk level compared to high-risk regions [5, 6]. However, when indicators of organized crime are examined, it is seen that Türkiye ranks among the higher positions globally and is classified within the “high-risk countries” category [7]. In this context, the distribution of crime by type, its intensity, and its changes over time in Türkiye are thought to play a determining role in shaping individuals’ perceptions of crime and fear of crime within society.

Fear of crime, an important manifestation of the phenomenon of crime, is a multidimensional construct that encompasses individuals’ perceptions of the likelihood of becoming a victim of crime, as well as the emotional, cognitive, and behavioral responses accompanying these perceptions [8, 9]. Moreover, fear of crime is emphasized not merely as an emotional response but as a complex construct encompassing risk perception, avoidance behaviors, and environmental evaluations [10]. In this respect, fear of crime is thought to be shaped not only by objective crime rates but also by how individuals perceive and interpret their environment. In this context, at the individual level, fear of crime is associated with negative outcomes such as decreased quality of life, impaired psychological well-being, and social isolation. At the societal level, it reduces the use of public spaces, limits social interaction, and contributes to the weakening of social ties [11]. Furthermore, fear of crime is reported to directly influence individuals’ everyday decisions and to have stronger effects particularly on disadvantaged groups [12]. In contrast, another prominent finding is that fear of crime often does not correspond directly to actual crime rates [9]. This discrepancy is thought to arise largely from the information sources to which individuals are exposed and how they interpret this information. In particular, the intensive presentation of crime and violence in national and social media increases individuals’ risk perceptions and reinforces their sense of danger [13]. Additionally, crime-related content in the media may lead individuals to feel more vulnerable and to perceive crime as something that can be encountered in real life. It is also noted that fear of crime further increases when such content aligns with individuals’ personal experiences [14]. In this context, the anthropological, legal, sociological, and psychological multidimensional and context-sensitive nature of fear of crime makes its accurate measurement critical.

A review of the literature shows that some fear-of-crime scales assess five types of crime [15], others examine three types [16, 17], and one evaluates only a single type of crime [18]. However, it has been reported that measuring fear related to different types of crime does not fully capture the emotional dimension of fear of crime and fails to represent the construct in its entirety [19]. In Türkiye, there are also studies aimed at measuring psychological constructs related to crime [20, 21]; however, existing scales appear to largely focus on criminal behavior and crime-related cognitions.

In light of this information, the limited number of measurement tools that directly and multidimensionally assess fear of crime within the Turkish cultural context [22] indicates a significant gap in the literature. In this regard, the aim of the present study is to adapt the Fear of Crime Scale developed by Etopio and Berthelot (2022) [19] into Turkish and to examine its validity and reliability.

## Methods

### Design

This study employed a cross-sectional methodological design to evaluate the psychometric properties of the Fear of Crime Scale. The study was conducted in accordance with guidelines aimed at strengthening the reporting of observational studies.

### Population and sample

The population of this study consisted of adult individuals aged 18–65 living in Turkey. Since the study aimed to assess the level of anxiety related to exposure to crime among adults, the population encompassed the general adult population. In determining the sample size, the commonly recommended rule of “5–10 times the number of items” in scale development studies was taken into account [23]. In addition to the commonly recommended participant-to-item ratio, the sample size was considered adequate based on recommendations suggesting that samples of 200–300 participants are generally sufficient for factor analytic procedures and that larger samples provide more stable factor loadings and more generalizable results. Furthermore, the final sample size substantially exceeded commonly recommended thresholds for factor analytic procedures, supporting the stability and robustness of the CFA results [23].

Accordingly, for the 10-item Fear of Crime Scale, the minimum sample size was calculated as 100; however, a larger sample was reached to increase the power of the analyses. The sample of the study consisted of a total of 849 adult individuals who voluntarily agreed to participate and met the inclusion criteria. This sample size was sufficient for conducting factor analyses reliably and allowed for a more comprehensive and robust examination of the scale structure. To evaluate test–retest reliability, paired measurements from a sufficient number of participants are required, and at least 30 pairs of data are recommended [24]. In the present study, test–retest reliability was assessed with 55 participants over a two-week interval.

The study data were collected between December 2025 and March 2026 using an online survey administered through Google Forms. The survey link was distributed via social media platforms, including Instagram, WhatsApp, Facebook, and X. A total of 918 responses were initially received. The inclusion criteria were being 18 years of age or older, being able to read and write in Turkish, and voluntarily agreeing to participate. The exclusion criteria included having a history of neurocognitive disorders or intellectual disability, responses containing at least one missing item, failure on any of the three attention-check items, completion times shorter than 5 min, and multivariate outliers identified using Mahalanobis distance. Three attention-check items were embedded in the questionnaire (e.g., “Please select ‘Agree’ for this item”, “Please select ‘Strongly Agree’ for this item”, and “Please select ‘Disagree’ for this item”). During data screening, 20 responses with missing data, 21 responses with incorrect answers to the attention-check items, 17 responses completed in less than 5 min, and 11 multivariate outliers identified using Mahalanobis distance were excluded. Following the data screening process, the final sample consisted of 849 participants.

### Measures

Data were collected using an Information Form, the Turkish Fear of Crime Scale developed by Ferraro (1995) and adapted by Kul (2009), the Fear of Crime Scale developed by Etopio and Berthelot (2022), and the Life Satisfaction Scale. The Turkish Fear of Crime Scale was used to assess convergent validity, whereas the Life Satisfaction Scale was used to assess discriminant validity.

### Information form

The Personal Information Form prepared by the researcher consists of questions aimed at determining the participants’ sociodemographic characteristics and their experiences related to crime. The form includes questions on variables such as age, gender, marital status, education level, employment status, income level, place of residence, and prior exposure to crime [19, 22].

### Fear of Crime Scale

The Fear of Crime Scale used in this study was developed by Ferraro (1995) to determine individuals’ levels of fear regarding different types of crime and was adapted into Turkish by Kul (2009) [22, 25]. The scale consists of statements related to various crime situations, and participants are asked to evaluate their level of fear for each situation. It includes items on different types of crime such as fraud, theft, sexual assault, being killed, robbery, and snatching. Each item is rated on a five-point Likert scale ranging from “1 = Not afraid at all” to “5 = Very afraid.” The scale consists of 11 items and has a unidimensional structure. Total scores range from 11 to 55, and there are no reverse-scored items or cut-off points. Higher total scores indicate higher levels of fear of crime. Regarding reliability, the Cronbach’s alpha coefficient was reported as high in the original study (α = 0.93) [22], and it was calculated as 0.94 in the present study.

### Life satisfaction scale

The Life Satisfaction Scale used in this study was developed by Diener et al. (1985) to determine individuals’ overall evaluations of their lives and was adapted into Turkish by Dağlı and Baysal (2016) [26, 27]. The scale has a unidimensional structure and consists of a total of 5 items. The items are rated on a five-point Likert scale ranging from “1 = Strongly disagree” to “5 = Strongly agree.” The total scores that can be obtained from the scale range between 5 and 25. There are no reverse-scored items or cut-off points in the scale. Higher total scores indicate higher levels of life satisfaction. Regarding reliability, the Cronbach’s alpha coefficient was reported as 0.88 in the adaptation study [27], whereas it was found to be 0.91 in the present study.

### Fear of Crime Scale (Adapted scale used in the present study)

The Fear of Crime Scale used in this study was developed by Etopio and Berthelot (2022) to measure individuals’ emotional responses to crime [19]. The scale consists of statements aimed at evaluating individuals’ emotional experiences such as fear, anxiety, worry, and uneasiness related to crime, and participants are asked to indicate their level of agreement with each statement. The scale has a unidimensional structure and consists of a total of 10 items. Items are rated on a six-point Likert scale ranging from “1 = Not at all true for me” to “6 = Completely true for me.” The scale score is calculated by taking the average of the item scores. There are no reverse-scored items or cut-off points in the scale. Higher scores indicate higher levels of fear of crime. Regarding reliability, the Cronbach’s alpha coefficient was found to be high in the original development study (α = 0.94) [19], and this value was also calculated as 0.94 in the present study.

### Procedures

Permission to evaluate and use the Turkish version of the Fear of Crime Scale was obtained via email from the corresponding author of the original scale. In assessing the psychometric properties of the Turkish adaptation, the principles established by the World Health Organization [28], the COSMIN [29], and the International Test Commission [30] were followed.

#### Stage 1 – Translation

The translation–back translation method was used to examine the linguistic properties of the scale. Accordingly, the scale was translated into Turkish by three independent linguists whose native language is Turkish, who are proficient in English, and who are familiar with the relevant scientific terminology.

#### Stage 2 – Synthesis

The three translated texts were compared in terms of semantic, conceptual, grammatical, and contextual aspects by the researchers, the translation team, and a Turkish language expert. Consensus was reached on each item, and the first Turkish draft of the scale was created; inter-translator agreement for the items was found to be above 95%.

#### Stage 3 – Back translation

The Turkish draft was translated back into English by two independent translators whose native language is English, who are proficient in Turkish, and who were not familiar with the original scale. The researchers and the back-translation team compared the two English versions, created a common draft, and confirmed consistency with the original scale items.

#### Stage 4 – Expert panel

After ensuring linguistic equivalence, the expert panel stage was conducted to evaluate the content validity of the scale. In this context, the Expert Evaluation Form, which included the original and Turkish draft forms, was sent via email to 10 faculty members who are experts in psychiatric nursing, sociology of crime, and psychology of crime. The experts were asked to evaluate each item according to the Davis technique and to provide feedback. As a result of the expert evaluations, the total Content Validity Index (CVI) was calculated as 0.981, and item-level CVI values ranged between 0.86 and 1.00.

#### Stage 5 – Pilot testing and cognitive review

Following the expert panel, the Turkish draft was administered to 30 adult individuals selected through purposive sampling outside the main sample in order to assess the clarity of the scale. During the application, participants were asked whether there were any difficult-to-understand expressions, and their suggestions, if any, were recorded.

#### Stage 6 – Final version and documentation

Since the participants found the items to be understandable, no revisions were deemed necessary, and the final version of the scale was approved.

#### Stage 7 – Main application

The main study data were collected at a single time point through an online survey administered via Google Forms. The data collection tools were completed by 794 participants who met the inclusion criteria, and completion of the questionnaires took approximately 20–30 min. To assess temporal stability (test–retest reliability), an additional sample of 55 participants was recruited separately and completed the Fear of Crime Scale twice at a two-week interval through face-to-face administration. The final version of the scale items is presented in the supplementary file (Supplementary File 1).

### Ethical considerations

This study was approved by the Social and Human Sciences Ethics Committee of Bandırma Onyedi Eylül University (Meeting no: 2025-11, Date of decision: 27/11/2025). Since the study was conducted in a community sample, institutional permission was not required. All participants were informed in detail about the study, and written informed consent was obtained. It was clearly stated that participation was entirely voluntary and that participants could withdraw at any stage without facing any consequences. Throughout the study, participants’ identities were kept confidential, and the collected data were used solely for scientific purposes.

### Data analysis

Statistical analyses were conducted by an independent biostatistician using SPSS 26.0 and AMOS 26.0 software. Because preliminary assessments indicated departures from multivariate normality, parameter estimates in the CFA were obtained using the Asymptotically Distribution-Free (ADF) estimation method. Sociodemographic characteristics of the participants were summarized using frequency (n), percentage (%), and mean ± standard deviation (X̄ ± SD). The Content Validity Index (CVI) based on the Davis technique was calculated to assess content validity. To evaluate construct validity, Confirmatory Factor Analysis (CFA) was performed, and parameter estimates were obtained using the ADF approach within the framework of structural equation modeling. Model fit was evaluated using χ²/df, GFI, AGFI, RMSEA, IFI, TLI, NFI, and SRMR indices, and AVE and CR values were calculated within the scope of convergent validity. The reliability of the scale was examined in terms of internal consistency, item analysis, and test–retest reliability. Cronbach’s alpha coefficient was calculated for internal consistency, and item–total score correlations were examined at the item level. To examine convergent and discriminant validity, the relationships between the Fear of Crime Scale and the Fear of Crime Scale and Life Satisfaction Scale were assessed using Pearson correlation coefficients. Temporal stability was assessed using the Intraclass Correlation Coefficient (ICC). A two-way mixed-effects model with absolute agreement was used, and single-measure ICC values [ICC(3,1)] were calculated. In all analyses, the level of statistical significance was set at *p* < 0.05.

## Results

### Participant’ profile

Of the participants, 68.1% were female, 68.2% were married, 47.5% were university graduates, and 41.9% were employed. Additionally, 75.6% had a middle income level, and 33.7% were living in metropolitan areas. While 55% of the participants reported feeling safe, 70.7% stated that they had not previously been exposed to any crime (Table 1).

**Table 1:** Participants’ profile (*n* = 849)

Variables	Number (*n*)	Percentage (%)
Age (years; X ± SD: 27.90 ± 11.23; min.–max.: 18–65)		
Gender		
Male	271	31.9
Female	578	68.1
Marital Status		
Married	579	68.2
Single	270	31.8
Educational Status		
Literate	24	2.8
Primary School	37	4.4
Middle School	50	5.9
High School	304	35.8
University	403	47.5
Postgraduate	31	3.6
Employment Status		
Employed	356	41.9
Unemployed	178	21.0
Retired	114	13.4
Student	201	23.7
Perceived Income Level		
Low	172	20.3
Middle	642	75.6
High	35	4.1
Place of Residence		
Metropolitan	286	33.7
City	210	24.7
District	157	18.5
Town/Village	196	23.1
Level of Feeling Safe in the Neighborhood		
I do not feel safe at all	31	3.7
I do not feel safe	131	15.4
No opinion	136	16.0
I feel safe	467	55.0
I feel completely safe	84	9.9
Previous Experience of Being a Crime Victim		
Yes	249	29.3
No	600	70.7

X ± SD = Mean±Standard Deviation

### Validity analyses

**Fig. 1:**
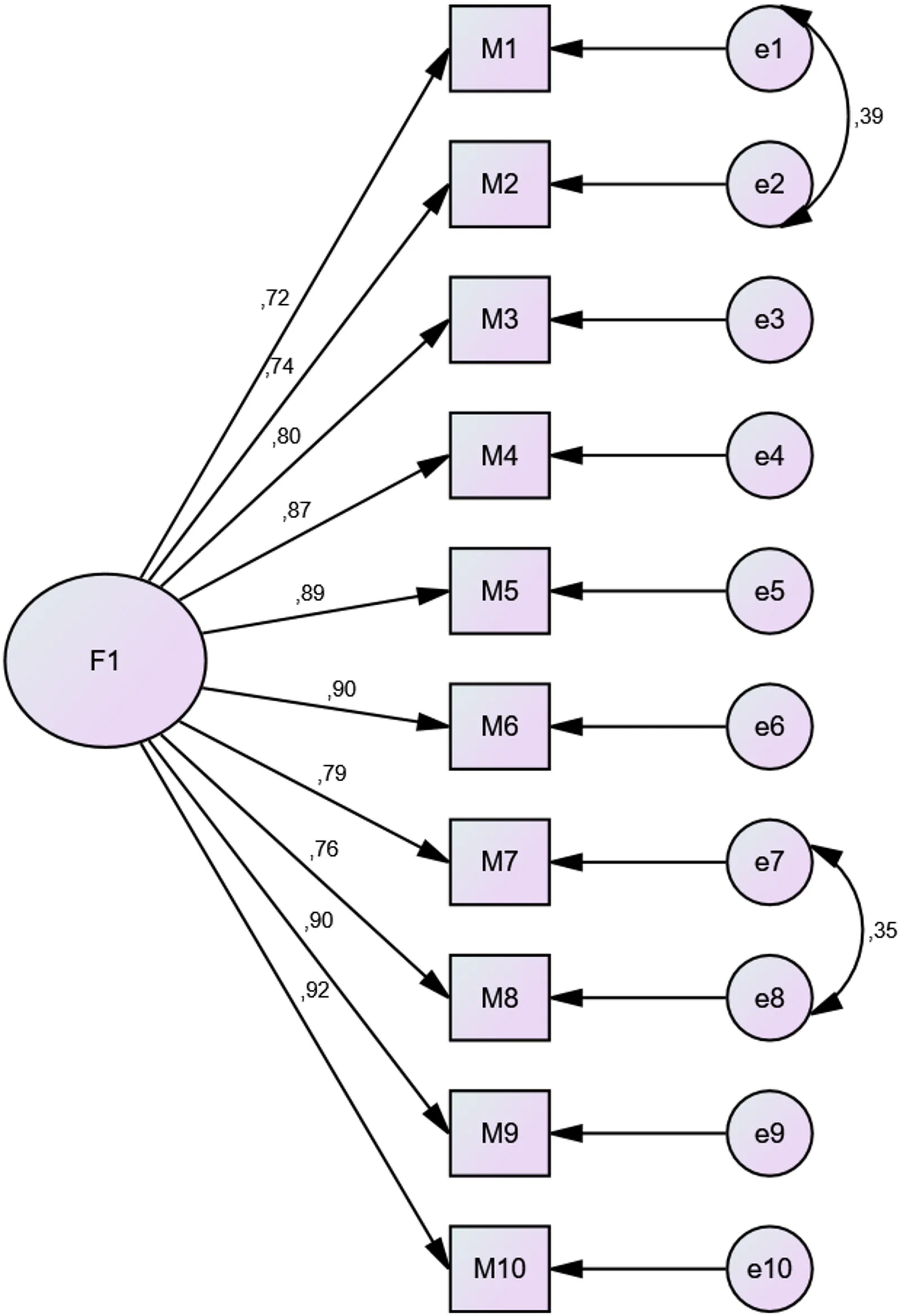
Confirmatory factor analysis factor loadings of the Fear of Crime Scale

Validity analyses of the scale included content validity, construct validity, and convergent/discriminant validity. Based on the opinions obtained from ten experts, the total CVI value calculated using the Davis technique in the present study was found to be 0.981. The CVI values for each item, based on expert evaluations, ranged between 0.86 and 1.00. Within the scope of confirmatory factor analysis, a first-order model was established to test the unidimensional structure of the scale, and the items were included in the model as indicators of this latent construct (Fig. 1). In the second stage of the model, parameter estimates were obtained using the ADF approach within the framework of structural equation modeling, and corresponding regression coefficients were calculated. The structure of the original scale, consisting of 10 items and a single factor, was confirmed, and two modifications were made (between items 1–2 and 7–8) to improve model fit. These modifications were not only statistically supported by modification indices but were also theoretically meaningful. Specifically, Items 1 and 2 both address perceived vulnerability to victimization, whereas Items 7 and 8 reflect cognitive preoccupation with personal safety and crime-related vigilance. In the final stage, model fit indices of the confirmatory factor analysis were evaluated. According to the findings, the model fit values were χ²/df = 4.90, GFI = 0.92, AGFI = 0.87, RMSEA = 0.08, IFI = 0.96, TLI = 0.93, NFI = 0.91, and SRMR = 0.05 (Table 2). Examination of the CFA results showed that the standardized factor loadings of the items ranged between 0.717 and 0.919. Additionally, AVE and CR values were found to be 0.69 and 0.95, respectively (Table 3).

**Table 2:** Confirmatory factor analysis fit indices

Fit Indices	Obtained Result	Perfect Fit	Acceptable Fit
χ²/df	4.90	0 ≤ χ²/df ≤ 3	3 ≤ χ²/df ≤ 5
GFI	0.92	0.90 ≤ GFI ≤ 1.00	0.85 ≤ GFI
AGFI	0.87	0.90 ≤ AGFI ≤ 1.00	0.85 ≤ AGFI
RMSEA	0.08	0.00 ≤ RMSEA ≤ 0.05	0.05 ≤ RMSEA ≤ 0.08
IFI	0.96	0.95 ≤ IFI ≤ 1.00	0.90 ≤ IFI
TLI	0.93	0.95 ≤ TLI ≤ 1.00	0.90 ≤ TLI
NFI	0.91	0.95 ≤ NFI ≤ 1.00	0.90 ≤ NFI
SRMR	0.05	0.00 ≤ SRMR ≤ 0.05	0.05 ≤ SRMR ≤ 0.08

**Table 3:** Standardized factor loadings and convergent validity indicators for the Fear of Crime Scale

Items	Factor	SE	CR	*p*	Standardized Factor Loading
Item 1	F1	--	--	--	0.717
Item 2	F1	0.040	25.911	< 0.001	0.738
Item 3	F1	0.045	24.746	< 0.001	0.800
Item 4	F1	0.052	23.570	< 0.001	0.872
Item 5	F1	0.054	22.838	< 0.001	0.892
Item 6	F1	0.057	22.802	< 0.001	0.900
Item 7	F1	0.053	19.848	< 0.001	0.792
Item 8	F1	0.053	18.460	< 0.001	0.764
Item 9	F1	0.055	23.978	< 0.001	0.898
Item 10	F1	0.056	24.154	< 0.001	0.919

Average Variance Extracted (AVE) = 0.69; Composite Reliability (CR) = 0.95

As part of the convergent and discriminant validity analyses, the Fear of Crime Scale demonstrated a significant positive correlation with the Fear of Crime Scale (*r* = 0.532, *p* < 0.001) and a significant negative correlation with the Life Satisfaction Scale (*r* = − 0.240, *p* = 0.041) (Table 4).

**Table 4:** Convergent and discriminant validity

Scale		Fear of Crime Scale Total (*r*)
Fear of Crime Scale	r	0.532
	p	< 0.001
Life Satisfaction Scale	r	-0.240
	p	0.041

*r* Pearson’s correlation coefficient

### Reliability analyses

The reliability of the scale was examined within the framework of internal consistency, item analysis, and test–retest reliability. In this context, the Cronbach’s alpha coefficient was calculated as 0.94. According to the results of the item analysis, the correlations between the items and the total score ranged between 0.616 and 0.842 (Table 5).

**Table 5:** Item-total correlations and cronbach alpha coefficients if item deleted for the Fear of Crime Scale

Items	Mean	SD	Item-Total Correlation	Cronbach Alpha if Item Deleted	Total Cronbach Alpha Coefficient
Item 1	4.09	1.68	0.616	0.942	0.94
Item 2	3.73	1.67	0.686	0.938	
Item 3	3.69	1.66	0.718	0.937	
Item 4	3.33	1.67	0.804	0.933	
Item 5	3.09	1.65	0.836	0.931	
Item 6	3.09	1.69	0.842	0.931	
Item 7	2.73	1.60	0.733	0.936	
Item 8	2.43	1.57	0.691	0.938	
Item 9	3.31	1.74	0.820	0.932	
Item 10	3.35	1.73	0.839	0.931	

In the test–retest analysis conducted to determine the temporal stability of the scale, the single-measure ICC [ICC(3,1)] based on a two-way mixed-effects model with absolute agreement was calculated as 0.927 (95% CI: 0.901–0.960) (Table 6).

**Table 6:** ICC and 95% confidence interval values for test-retest reliability of the Fear of Crime Scale

Scale	ICC	95% CI Lower	95% CI Upper	*p*
Fear of Crime Scale Total Score	0.927	0.901	0.960	< 0.001

*ICC* Intraclass Correlation Coefficients

## Discussion

The findings of this study, which was conducted to evaluate the psychometric properties of the Turkish version of the Fear of Crime Scale, are discussed based on validity and reliability analyses. A review of the literature revealed no adaptation studies evaluating the psychometric properties of the scale developed by Etopio and Berthelot (2022) in different languages or cultural contexts [19]. This suggests that the findings obtained for the Turkish version of the scale provide an original contribution to the literature.

### Validity analysis

In this study, the Content Validity Index (CVI) was examined to determine the content validity of the scale items based on expert opinions. All items included in the scale were evaluated by experts, and these evaluations were used in calculating the CVI. According to commonly accepted criteria, CVI values above 0.79 are considered acceptable, values between 0.70 and 0.79 are considered questionable, and values below 0.70 are considered unacceptable [31]. CVI values above 0.90 indicate an excellent level of agreement among experts [32]. In our study, the overall CVI was found to be 0.981, and item-level CVI values ranged between 0.86 and 1.00. These findings indicate that the Turkish version of the scale has a high level of content validity. In the original scale development study, content validity was not assessed using the CVI. Instead, content validity was established through qualitative interviews, cognitive evaluations, and expert opinions, and no numerical content validity index was reported [19]. This suggests that content validity can be assessed using different methods. In this context, it can be argued that the objective and quantifiable findings obtained through the evaluation of content validity using the CVI in our study are consistent with the content validity established through qualitative methods in the original study. The high CVI values obtained in the present study indicate that the scale items adequately represent the construct intended to be measured. Accordingly, it can be stated that the Turkish version of the scale has strong content validity.

In the present study, Confirmatory Factor Analysis (CFA) was conducted to evaluate the construct validity of the Turkish-adapted scale. In this context, the preservation of the original single-factor structure was tested, and the 10-item unidimensional structure of the scale was confirmed. According to the CFA results, the factor loadings of the 10 items ranged from 0.717 to 0.919. While factor loadings above 0.50 are considered practically significant, loadings above 0.70 are regarded as strong indicators of the latent construct [33]. Therefore, the high factor loadings obtained indicate that the items strongly represent the corresponding factor and that the scale has a robust factor structure. In the original scale development study, both Exploratory Factor Analysis (EFA) and Confirmatory Factor Analysis (CFA) were conducted to assess construct validity. The EFA results revealed a single-factor structure consisting of ten items, which explained 63.75% of the total variance, with factor loadings ranging from 0.622 to 0.865. The CFA findings obtained in the original study also supported the unidimensional structure of the scale and confirmed the EFA results, showing that the standardized factor loadings ranged between 0.715 and 0.888 [19]. Taken together, these findings suggest that the scale has a unidimensional structure and that the items adequately and strongly represent the construct of ‘fear of crime.’ The CFA findings obtained in the present study also support this structure, indicating that construct validity has been established for the Turkish version of the scale. Furthermore, the similarity between the factor loadings obtained in the present study (0.717–0.919) and those reported in the original scale development study (0.715–0.888) suggests that the underlying construct of fear of crime was preserved across cultural contexts. In the present study, EFA was not performed because the scale had an established and theoretically supported unidimensional structure in the original scale development study. Since the primary aim of the present study was to evaluate whether the original factor structure could be replicated in the Turkish cultural context, construct validity was assessed through CFA. CFA is considered an appropriate approach in adaptation studies when there is a predefined factor structure to be tested. Nevertheless, future studies may further examine the dimensional structure of the Turkish version using both exploratory and confirmatory factor analytic approaches.

In the present study, model fit indices related to CFA were examined to evaluate the overall model fit of the scale. To assess construct validity, certain criteria for model fit must be met. Accordingly, the recommended threshold values for goodness-of-fit indices are χ²/df ≤ 5, RMSEA and SRMR ≤ 0.08, and AGFI, GFI, TLI, CFI, IFI, and NFI ≥ 0.90 [34–36]. Accordingly, considering the chi-square value (χ²/df = 4.90) obtained in the present study, together with the other fit indices (GFI = 0.92, AGFI = 0.87, RMSEA = 0.08, IFI = 0.96, TLI = 0.93, NFI = 0.91, and SRMR = 0.05), it can be concluded that the model demonstrates an overall acceptable and good level of fit. Therefore, our findings indicate that the model is consistent with the data and that the construct validity of the scale has been established. In the original scale development study, the CFA results were reported to be insufficient for some fit indices and acceptable for others (χ² = 228.94, *p* < 0.001; RMSEA = 0.124; SRMR = 0.053; CFI = 0.898) [19]. In contrast, the fit indices examined in the present study were generally found to be at acceptable and good levels. This finding may indicate that the Turkish version demonstrates a factor structure that is at least as stable as that reported in the original study.

In the present study, convergent validity was evaluated using the Average Variance Extracted (AVE) and Composite Reliability (CR), which were calculated as 0.69 and 0.95, respectively. Since the AVE exceeds 0.50 and the CR exceeds 0.60, these findings indicate that the scale has satisfactory convergent validity [37]. To provide a more comprehensive assessment of convergent validity, the relationship between the scale and another instrument measuring fear of crime was examined, and a significant positive association was found between the two scales (*r* = 0.532, *p* < 0.001). This finding indicates that the expected relationship was observed between instruments measuring similar constructs and supports convergent validity. In the original study, convergent validity of the scale was examined through correlation analyses with different scales measuring fear of crime, and the correlations with these scales were reported to range between 0.640 and 0.718 [19]. These findings also provided evidence for convergent validity in the original version of the scale. In addition, discriminant validity was evaluated using theoretically distinct constructs, and correlations ranged from − 0.054 to 0.444. Although some relationships were weak or non-significant, the overall pattern of findings supported the distinctiveness of the fear of crime construct from theoretically unrelated variables [19]. Taken together, the findings of the present study are consistent with those reported in the original scale development study and support both the convergent and discriminant validity of the Turkish version of the scale. Moreover, unlike the original study, convergent validity was evaluated not only through correlation analyses but also through AVE and CR values, providing additional quantitative evidence for the construct validity of the scale. Discriminant validity was also supported by the significant negative correlation observed between the Fear of Crime Scale and the Life Satisfaction Scale. This finding was theoretically expected and suggests that higher levels of fear of crime may be associated with lower levels of life satisfaction.

### Reliability analysis

In the present study, the reliability of the scale was evaluated using internal consistency (Cronbach’s alpha), item analysis, and test–retest reliability methods. The Cronbach’s alpha coefficient was found to be 0.94, while item analysis results showed that the correlations between each item and the total score ranged from 0.616 to 0.842. Test–retest reliability, assessed by re-administering the scale over a specified time interval and calculating the intraclass correlation coefficient (ICC), was found to be 0.927. According to generally accepted criteria, a Cronbach’s alpha coefficient of 0.70 or higher indicates acceptable internal consistency, whereas values of 0.80 or higher indicate good to excellent internal consistency for practical applications [38]. Accordingly, Cronbach’s alpha coefficient of 0.94 obtained in the present study substantially exceeds the recommended thresholds for acceptable and good internal consistency, indicating excellent reliability [24, 33]. ICC values are interpreted based on the 95% confidence interval as moderate (0.50–0.74), good (0.75–0.90), and excellent (> 0.90) [39]. In line with these criteria, the findings obtained in the present study indicate that the Turkish version of the scale demonstrates a high level of reliability in terms of internal consistency (Cronbach’s α and item–total correlations) and temporal stability (ICC). In the original study conducted by Etopio and Berthelot (2022), the reliability of the scale was evaluated using Cronbach’s alpha coefficient (α = 0.945); however, no analysis regarding temporal stability was reported. The nearly identical Cronbach’s alpha coefficients obtained in the original study (α = 0.945) and the present study (α = 0.94) provide further evidence for the cross-cultural stability and consistency of the scale. In the original study conducted by Etopio and Berthelot (2022), the reliability of the scale was evaluated using Cronbach’s alpha coefficient (α = 0.945); however, no analysis regarding temporal stability was reported [19]. In the present study, the reliability of the scale was examined in greater detail by including the test–retest method, and temporal stability was also demonstrated through this approach. 

## Limitations

The cross-sectional design of this study limits the ability to draw causal inferences based on the findings and constitutes one of the main limitations of the study. Additionally, the use of self-report measures for data collection may have introduced measurement biases such as social desirability and recall bias, and may have restricted the findings to participants’ self-reports. The fact that the study sample consisted of volunteer individuals living in Türkiye, and that a large proportion of the participants were female and of middle income, may limit the generalizability of the findings. In addition, the use of online data collection methods may have led to the exclusion of individuals without internet access. Additionally, exploratory factor analysis was not conducted in the present study.

## Implications for practice

The findings obtained from this study indicate that the Turkish version of the Fear of Crime Scale is a valid and reliable measurement tool. Owing to its brief, practical, and reliable structure, the scale can be used by criminology researchers and mental health professionals working in the field of crime psychology. The scale is considered to be useful for assessing fear of crime among individuals from different segments of society and for examining its relationship with various variables such as psychological well-being. Furthermore, its application across diverse cultural contexts and socioeconomic levels may contribute to a better understanding of the social distribution of fear of crime. In this regard, the findings obtained from the scale may also contribute to the development of intervention strategies aimed at coping with fear of crime. Although CFA supported the original factor structure, future studies may further examine the dimensional structure of the Turkish version using both exploratory and confirmatory factor analytic approaches. In addition, future research may evaluate the psychometric properties of the scale in more diverse and representative populations, including individuals from different age groups, socioeconomic backgrounds, educational levels, and geographical regions. Such studies may provide further evidence regarding the generalizability and applicability of the scale across different segments of society. Future studies may also investigate measurement invariance across key demographic groups (e.g., gender and age) to further establish the comparability and generalizability of the scale across different populations.

## Conclusion

The findings of this study, which evaluated the psychometric properties of the Turkish version of the Fear of Crime Scale, indicate that the scale can be used as a valid and reliable measurement tool. The results obtained from validity and reliability analyses confirmed the unidimensional structure of the original scale and demonstrated that the items adequately represent the construct intended to be measured. Consequently, the Turkish version of the Fear of Crime Scale can be considered a valid and reliable instrument for assessing emotional responses related to fear of crime among adult individuals.

## Supplementary Information

Supplementary Material 1.

## Data Availability

The datasets used and/or analysed during the current study are available from the corresponding author on reasonable request.
