# Supplementary material for: Psychometric evaluation of the Turkish version of the Fear of Crime Scale
Source: BMC Psychol. 2026 Jul 2;14:1231. doi: 10.1186/s40359-026-05110-3 (PMC13495293; doi:10.1186/s40359-026-05110-3)
Supplement: Supplementary file 1 [file 40359_2026_5110_MOESM1_ESM.docx]

**Appendix 1.** English Version of the Fear of Crime Scale (Corresponding to the Turkish Form)

|  | | 1 = very untrue for me | 2 = untrue for me | 3 = somewhat untrue for me | 4 = somewhat true for me | 5 = true for me | 6 = very true for me |
| --- | --- | --- | --- | --- | --- | --- | --- |
| 1 | I am afraid that a crime will be committed against me. |  |  |  |  |  |  |
| 2 | I feel vulnerable to becoming a victim of a crime committed against me. |  |  |  |  |  |  |
| 3 | The possibility that a crime may be committed against me is always at the back of my mind. |  |  |  |  |  |  |
| 4 | The possibility of a crime being committed against me causes me emotional stress. |  |  |  |  |  |  |
| 5 | The possibility of a crime being committed against me makes me worried in my daily life. |  |  |  |  |  |  |
| 6 | I cannot relax because of the possibility of a crime being committed against me. |  |  |  |  |  |  |
| 7 | My mind is constantly occupied with protecting myself from a crime that may be committed against me. |  |  |  |  |  |  |
| 8 | I feel paranoid about my personal safety due to the possibility of a crime being committed against me. |  |  |  |  |  |  |
| 9 | Knowing that a crime may be committed against me makes me uncomfortable. |  |  |  |  |  |  |
| 10 | The possibility of a crime being committed against me makes me feel restless. |  |  |  |  |  |  |

**Appendix 2.** Turkish Version of the Fear of Crime Scale (Adapted Form)

|  | | 1 = Benim için hiç doğru değil | 2 = Benim için doğru değil | 3 = Benim için biraz doğru değil | 4 = Benim için biraz doğru | 5 = Benim için doğru | 6 = Benim için tamamen doğru |
| --- | --- | --- | --- | --- | --- | --- | --- |
| 1 | Bana karşı bir suç işlenmesinden korkuyorum. |  |  |  |  |  |  |
| 2 | Bana karşı işlenebilecek bir suçun kurbanı olma konusunda kendimi savunmasız hissediyorum. |  |  |  |  |  |  |
| 3 | Bana karşı bir suç işlenme ihtimali her zaman aklımın bir köşesindedir. |  |  |  |  |  |  |
| 4 | Bana karşı bir suç işlenme ihtimali bende duygusal stres yaratıyor. |  |  |  |  |  |  |
| 5 | Bana karşı bir suç işlenme ihtimali günlük yaşamımda beni endişelendiriyor. |  |  |  |  |  |  |
| 6 | Bana karşı bir suç işlenme ihtimali nedeniyle rahat hissedemiyorum. |  |  |  |  |  |  |
| 7 | Zihnim sürekli bana karşı işlenebilecek bir suçtan kendimi korumakla meşgul. |  |  |  |  |  |  |
| 8 | Bana karşı bir suç işlenme ihtimali yüzünden kendi güvenliğimle ilgili paranoyak hissediyorum. |  |  |  |  |  |  |
| 9 | Bana karşı bir suç işlenme ihtimalini bilmek beni rahatsız ediyor. |  |  |  |  |  |  |
| 10 | Bana karşı bir suç işlenme ihtimali beni huzursuz ediyor. |  |  |  |  |  |  |
